# Addressing Psychosocial Factors in Cognitive Impairment Screening from a Holistic Perspective: The DeCo-Booklet Methodology Design and Pilot Study

**DOI:** 10.3390/ijerph191912911

**Published:** 2022-10-09

**Authors:** Cristina García, Lucrecia Moreno, Mónica Alacreu, Francisco J. Muñoz, Luis A. Martínez

**Affiliations:** 1 Cátedra DeCo MICOF-CEU UCH, Universidad Cardenal Herrera-CEU, 46115 Valencia, Spain; 2 Department of Pharmacy, Universidad Cardenal Herrera-CEU, CEU Universities, 46115 Valencia, Spain; 3 Community Pharmacist, 02161 Albacete, Spain; 4 Embedded Systems and Artificial Intelligence Group, Universidad Cardenal Herrera-CEU, 46115 Valencia, Spain

**Keywords:** cognitive impairment, heathy aging, psychosocial factors, pharmacy, purpose in life, meaning in life

## Abstract

Cognitive impairment (CI), an intermediate phase between the decline in physiological cognition and dementia, is known to be mediated by a variety of risk and protective factors, with age being the most influential of these. The multifactorial nature of CI and the worldwide phenomenon of an aging population makes decoupling old age from disease through the concept of healthy aging (HA) a matter of major interest. Focusing on psychosocial variables and psychological constructs, here we designed and piloted a data collection booklet (DeCo-B) to assess CI and HA from a holistic perspective. The DeCo-B comprises six sections: sociodemographic factors, CI, meaning in life, psychosocial factors, health problems, and lifestyle. The estimated prevalence of CI and HA in our cohort were 24.4% and 6.6%, respectively. Spearman correlations mainly identified pairwise associations between the meaning in life domains and psychosocial variables. Moreover, age, marital status, purpose in life, resilience, chronic pain, cognitive reserve, and obstructive sleep apnea were significantly associated with an increased risk of CI. Our results showed that DeCo-B is a suitable tool for researching how modifiable risk and protective factors influence cognitive status. The complex interrelationships between variables should be further investigated and, for practical reasons, the questionnaire should be optimized in future work.

## 1. Introduction

Cognitive impairment (CI) is an intermediate stage between a physiological decline in cognition associated with aging and conditions severe enough to interfere with social abilities and daily functioning, such as dementia. Therefore, CI marks the boundary between healthy aging (HA) and the onset of neurodegenerative diseases, with Alzheimer’s disease (AD) being the most common form of dementia [1,2]. However, this line may be blurred because the evolution of CI to dementia is a reversible process that depends on many factors [3]. Nonetheless, given the lack of effective pharmacological treatments [4], the increased risk of progression observed in cognitively impaired patients makes early diagnosis the strategy of choice, through screening coupled with a combination of medical and lifestyle interventions.

Several risk factors (RFs) are strongly linked to CI. In 2019, the World Health Organization (WHO) published guidelines for modifiable RFs to help reduce cognitive decline, including the control of different pathologies (e.g., hypertension, depression, and diabetes) and lifestyle habits (e.g., physical activity and nutrition) [5]. The 2020 report from the Lancet Commission on Dementia Prevention, Intervention, and Care estimated that up to 40% of dementias might be preventable by modifying only 12 RFs, including education levels, a history of head injury, and air pollution. However, age, a non-modifiable RF, still ranks as the greatest contributor to the prevalence of CI [6].

Population aging is a global phenomenon and the world population aged over 65 years is expected to reach 16% by 2050 [7]. This huge increase, driven by rising life expectancies and decreasing levels of fertility, will undoubtedly impact on the prevalence of dementias, making it even more important to decouple aging from disease. In Spain, this is especially relevant in rural areas which tend to have more aged populations and in which depopulation and barriers to accessing health services can lead to an even more complex scenario [8].

The WHO defines HA as the process of developing and maintaining functional abilities that enable well-being and helping to avoid disease in older age [9]. Although the concept of HA is currently understood as a lifelong process driven by intrinsic and environmental factors [10], the WHO’s definition agrees with Rowe and Kahn’s model [11,12] which considers successful aging as: (i) the absence of disease and disability; (ii) normal cognition and physical function; and (iii) engagement in social and productive activities. According to point (ii) of this definition, maintaining a good cognitive function is necessary to consider that HA is achieved. Hence, the ageing process can be modulated through exposure to CI risk and protective factors. In this respect, a number of these factors have been recently described to mostly overlap with several HA domains [13]. Among them, psychosocial variables (e.g., satisfaction with life, resilience, social networks, and loneliness) have been found to be as relevant as biological markers in the assessment and quantification of the so-called healthy aging phenotype [14]. Moreover, the construct of psychological wellbeing has also drawn the attention of researchers given its relationship to better health and physical and cognitive status [15,16].

Meaning in life (MiL) is defined as the ability to endow content and significance to one’s life. This concept has evolved since it was first proposed and is now accepted to encompass three different dimensions: cognitive (coherence); motivational (purpose); and affective (engagement). Each facet fulfils different human functions and should be assessed and evaluated separately. Coherence comprises environment comprehension, stimuli management, and the use of available resources to interact with media. The term purpose denotes the feeling that one’s life is moving through a definite path in pursuit of a goal, while engagement represents the degree of individual satisfaction and fulfilment in life [17,18,19,20]. The relationship between MiL, or its individual dimensions, and CI has become an interesting topic of research, as several papers have shown that (i) MiL and cognitive health outcomes may be linked [21,22,23] and (ii) MiL influences RFs for cognitive decline [24,25,26]. Keeping this in mind, it seems advisable to evaluate the influence of psychosocial variables on CI in two different ways: (i) as modifiable risk and protective factors per se and (ii) as a tool to influence non-modifiable RFs, such as age.

Therefore, multifactorial processes such as CI and HA are influenced by multiple variables covering different fields. Assessing this wide range of factors and evaluating their relationships with CI could provide valuable information on the effect of the exposure to a given factor. Based on this data, we could (i) design specific prevention strategies and (ii) anticipate a higher risk of cognitive alterations. As a preliminary approach, this could be achieved by screening a target population using a battery of objective and validated tests to identify those who might be affected by the early stages of cognitive alterations. This group could then undergo an in-depth clinical–neurological assessment. Thus, approaching CI from a holistic perspective could anticipate detection of cognitively impaired patients in their own environments and their referral to confirm the diagnosis. In fact, strategies based on community are known to increase the detection rate of patients with undiagnosed CI in the early stages of the disease, to improve its prognosis and its evolution [27].

Thus, the main goals of this current study were (i) to design a data collection booklet (DeCo-B) comprising a set of validated screening tests as a holistic approach designed to assess CI and HA through risk and protective variables and (ii) to perform a pilot study to test its usefulness for evaluating the risk and prevalence of CI and HA.

## 2. Materials and Methods

### 2.1. Bibliographic Review

We conducted a thorough literature review of the records indexed in the PubMed and Web of Science databases to identify tools and instruments used to evaluate how modifiable risk and protective factors might influence CI. Some scales were selected based on the previous experience of our research group or through recent literature reviews [27,28,29]. Because we aimed to ascertain whether the simultaneous evaluation of psychosocial variables, MiL, and cognitive status was achievable in face-to-face interviews, we focused our search on psychosocial factors and MiL assessment methods suitable for CI screening. The results of the review were used to select the most appropriate instrument for each category included in the DeCo-B.

The academic literature was searched from 2017 to 2022, considering only articles written in English, using the following keywords: (1) “cognitive impairment”, “cognitive decline”, “dementia”, “Alzheimer disease” and “cognition”; (2) “meaning in life”; (3) “sense of coherence”; (4) “purpose in life”; (5) “engagement with life”; (6) “resilience”; (7) “stress”; (8) “loneliness”; and (9) “social isolation”. The inclusion and exclusion criteria are shown in Table 1.

### 2.2. Data Collection Booklet Design

To collect information using a holistic approach, we designed an ad hoc DeCo-B comprising a selection of validated screening questionnaires and tests to assess our dependent variable (CI) and several risk and protective factors, which we classified into six categories: (i) sociodemographic factors; (ii) CI; (iii) MiL; (iv) psychosocial factors; (v) health problems; and (vi) lifestyle. Instruments to assess each variable were selected based on our bibliographic literature review, as outlined above. 

#### 2.2.1. Socio-Demographic Factors

Data on gender, age, marital status, and family history of dementia were collected during the interview.

#### 2.2.2. Cognitive Impairment

CI was assessed using the Spanish versions of three different methods: (i) the Memory Impairment Screen (MIS), a useful tool for detecting memory impairment through reading and subsequent free and facilitated recall of four words. The sensitivity for general dementia in the Spanish population was 74% and the specificity 96%; (ii) the Semantic Verbal Fluency Test (SVF), a psychological test in which interviewees recall as many words as possible from a given category (in this case, animals) in a determined period of time. The sensitivity for this test in Spain was 90% and the specificity 94% in a population with a poor educational level; and (iii) the Short Portable Mental State Questionnaire (SPMSQ) or Pfeiffer Test, a rapid test (suitable for illiterate individuals) which includes questions on orientation, memory, and attention. The sensitivity and specificity for CI was 85.7% and 79.3%, respectively [30,31,32]. A participant was considered cognitively impaired if they tested positive for at least one of these tests [27].

#### 2.2.3. Meaning in Life

The tools included in the DeCo-B for this factor were selected based on our literature review (see Section 3.1 and Table 2).

#### 2.2.4. Psychosocial Factors

The tools included in the DeCo-B for resilience, stress, loneliness, and social isolation were selected based on our literature review (see Section 3.1 and Table 2). We also included the Geriatric Depression Scale (GDS-5) and Patient Health Questionnaire-4 (PHQ-4) as described in Section 3.1 and Table 2, which we had already used in previous work [27,29].

#### 2.2.5. Health Problems

A pharmaceutical interview was conducted for each patient according to the Dader Method [33]. The Anatomical Therapeutic Chemical Code (ATC) [34] and the International Classification of Diseases (ICD-10) [35] were used for encoding. A parallel medication review was also carried out [36] that collected information concerning concordance, treatment lengths, dosage schedules, defined daily doses, posology, adherence, and common blood analysis parameters. Data were evaluated to identify any drug-related problems and negative outcomes related to medicines, which were then classified and quantified. Drug interactions were specifically investigated using the Spanish General Council of Pharmaceutical Associations Medicines Database [37] and CheckTheMeds^®^ [38]. Furthermore, cardiovascular risk was calculated according to the ERICE Scale [39]. Smoking habits, dependency, hearing loss, and chronic pain were also assessed [29].

#### 2.2.6. Lifestyle

Variables in this category (anthropometry, nutrition, cognitive reserve, physical activity, and sleep) was already assessed by our group in previous research [29].

### 2.3. Healthy Aging

The prevalence of HA was calculated as conceptualized by Rowe and Kahn: the absence of major illness (myocardial infarction/heart attack; cardiac failure; angina; osteoarthritis, arthritis, or rheumatism; chronic bronchitis, emphysema, or chronic obstructive pulmonary disease; diabetes; depression; anxiety; cerebral embolism/infarction; or malignant tumors/cancer), no daily living disability, adequate cognitive function, normal physical functioning, being ‘actively engaged’, and the absence of disease risk factors such as obesity and self-referred hypertension. Following these authors, we only considered those participants who met all these criteria as having aged healthily [40].

### 2.4. Population

A cross-sectional study was conducted from September 2021 to March 2022. The inclusion criteria for participation were non-institutionalized patients aged over 50 years. Exclusion criteria were patients diagnosed with AD or dementia at baseline or with a severe sensory, physical, or psychological disability preventing the interview from being carried out. The potential participants were offered the possibility of participation in the study alongside information about it. After checking for eligibility, a face-to-face appointment was scheduled. Once the signed informed consents of the patients were obtained, they were interviewed at appropriate facilities, always maintaining their confidentiality throughout the whole process.

The necessary sample size was calculated using the classical formula to perform unilateral contrasts between two independent means, determined by the quantitative scales selected for the DeCo-B and the groups of individuals with and without risk of CI. In this formula, the following parameters were taken: a significance level of 0.05, a power of 80%, a ratio between the groups of 1:3, and an estimation of the standard deviation of the scale. All the necessary sample sizes are smaller than the one obtained in the study (*n* = 213), except the one to contrast the MIL and psychosocial variables, which required at least 1.398 participants (difference between the mean values of 2 points and typical deviation of 13 points, approx.).

### 2.5. Statistical Analysis

The data collected in the DeCo-B during the interviews were stored in an ad hoc Excel^®^ file. An initial descriptive analysis was performed for all of the variables registered in the dataset. Numerical variables were described using means and standard deviations. Many of them were categorized using cut-off points provided in the academic literature. Thus, we applied a two-fold rank and qualitative analysis to these variables. The qualitative variables were described as the absolute number and percentages: n (%).

Because most of the variables were numerical, the Shapiro–Wilk test was used to test the normality of the data and the pairwise correlation was tested using Spearman’s rho coefficient and corresponding hypothesis testing. To determine the significance of the results we used the false positive rate as an adjusted *p*-value.

Finally, we examined the association between variables and CI compatible scores. To analyze these associations, appropriate hypothesis testing was employed (Wilcoxon rank-sum, Chi-squared, Fisher exact, and *t*-tests), depending on the characteristics of the variables involved. Logistic regression models were also created to assess the association between risk of CI and purpose in life (PiL), engagement with life (EwL), resilience, and chronic pain, all as a function of age. All the statistical analyses were completed using advanced statistics in R software (R-4.2.1, R Foundation for Statistical Computing, Vienna, Austria). The significance levels considered are detailed in the Results section where appropriate.

### 2.6. Ethical Approval

This study was approved by the Research Ethics Committee at the Universidad CEU Cardenal Herrera (approval no. CEI22/249) and by the Drugs Research Ethics Committee at Arnau de Vilanova Hospital (MOR-ROY-2018-013). In accordance with the Declaration of Helsinki, all of the participants gave their written informed consent to participation.

**Table 2:** Summary of the tools or scales used in each study.

Variable	Tools or Scale	No. of Items	Study
**Meaning in Life**			
Whole construct	Meaning in Life Questionnaire (MLQ)	10	McGee et al., 2017 [41] Aftab et al., 2019 [42]
Sense of coherence	**Orientation to Life Questionnaire** **(OLQ)**	**13**	Bartrés-Faz et al., 2018 [26] Macià et al., 2021 [43] Sutin et al., 2020 [23]
Purpose in life	**Purpose in life subscale of Ryff’s Well-Being Scale (PiL)**	**6**	Bartrés-Faz et al., 2018 [26] Macià et al., 2021 [43]
		7	Sutin et al., 2018 [44] Lewis et al., 2021 [45] Kim et al., 2019 [22]
		6 and 7	Sutin et al., 2021 [21]
	PiL scale from Ryff and Keyes’ Psychological Wellbeing Scale	10	Wingo et al., 2020 [46] Boyle et al., 2021 [47]
Engagement with life	**Engaged Living Scale** **(ELS)**	**16**	Bartrés-Faz et al., 2018 [26] Macià et al., 2021 [43]
**Psychosocial Factors**			
Resilience	**Brief Resilience Coping Scale (BRCS)**	**4**	Meléndez et al., 2018 [48]
	Connor–Davidson Resilience Scale (CD-RISC)	25	Eyre et al., 2017 [49]
Stress	**Perceived Stress Scale** **(PSS)**	**4**	Turner et al., 2017 [50] Feeney et al., 2018 [51]
		10	Elkana et al., 2020 [52]
		14	Katz et al., 2016 [53] Jiang et al., 2017 [54]
Loneliness	**UCLA Loneliness Scale**	**3**	Lara et al., 2019 [55] Jang et al., 2021 [56]
		11	Lee et al., 2021 [57]
		20	Kwon et al., 2017 [58] Gené-Badia et al., 2020 [59]
	De Jong Gierveld Loneliness Scale	6	Burholt et al., 2017 [60] Fung et al., 2019 [61] Evans et al., 2019 [62]
Social isolation	**Lubben Social Network Scale** **(LSNS)**	**6**	Burholt et al., 2017 [60] Evans et al., 2019 [62] Li et al., 2019 [63] Gené-Badia et al., 2020 [59] Jang et al., 2021 [56] Foong et al., 2021 [64]
		12	Siette et al., 2020 [65]
	Shankar Index	5	Lara et al., 2019 [55]

The tests or scales shown in bold were selected for use in the DeCo-B.

## 3. Results

### 3.1. Selection of the Tools to Assess Meaning in Life and Psychosocial Factors

After screening the articles retrieved by our initial search according to the exclusion criteria and eliminating duplicates, 29 references were selected in which the CI and psychosocial variables or MiL were simultaneously assessed. The resulting studies were analyzed in depth to select the tools most suitable for our research design, as summarized in Table 2 with references to the original sources. The tests selected for inclusion in the DeCo-B are marked in bold. Some references are repeatedly cited in Table 2 because the corresponding article detailed the assessment of several variables and, thus, used more than one instrument of interest. In our opinion, the association between the referenced study and the tool of interest can be more easily inferred in this way.

Regarding MiL, the Meaning in Life Questionnaire (MIQ) was discarded because it estimates MiL as a single construct. According to the bibliography and considering the evolution of the concept, we found it more reasonable to separately assess each dimension (the Sense of Coherence (SoC), PiL, and EwL) because there are suitable methods that allow this. Our search retrieved only one test for assessing the SoC and EwL; for the PiL, we selected the six items of the PiL subscale from Ryff’s Well-Being Scale, which had already been used to separately assess the three MiL dimensions.

In terms of psychosocial factors, and as a general criterion, we preferred short questionnaires for two reasons: (i) so we could design the DeCo-B so that the overall time required for its administration would be reasonable and (ii) to ensure that DeCo-B would encompass as many variables as possible. Thus, the shortest version of the Perceived Stress Scale, UCLA Loneliness Scale, and Lubben Social Network Scale were selected to assess stress, loneliness, and social isolation, respectively. Moreover, these versions were the most frequently used in Spanish population studies. Finally, based on the same selection approach as described above, we selected the Brief Resilience Coping Scale rather than the Connor Davidson Resilience Scale to evaluate resilience.

### 3.2. Data Collection Booklet and Pilot Study

Once the DeCo-B was designed using selected tools and scales from the bibliographic review, we collected complete data from a total of 213 interviews which incorporated a total of 31 variables from 23 validated tests. The DeCo-B structure (test references, number of items, cut-off scores, and score range for each scale) is shown in Table 3 (columns 1–3). For the sake of clarity, a descriptive analysis of the results obtained during the DeCo-B piloting phase (column 4) is shown together with the DeCo-B structure, including information on the test duration (column 5). The mean time required to administer the DeCo-B was 69 min.

#### 3.2.1. Sociodemographic Factors

The participants (*n* = 213) were aged 72.8 ± 11.2 years, were mainly female and married (61.2% and 65.6%, respectively), and only 16.0% had a family history of dementia.

#### 3.2.2. Cognitive Impairment

Among the participants, 79 (37.1%) reported a subjective memory complaint while 24.4% obtained results compatible with CI. The Pfeiffer questionnaire detected the highest rate of positive assessments.

#### 3.2.3. Meaning in Life

Because the scales selected to assess the MiL domains had no cut-off point, the data relating to SoC, PiL, and EwL had to be assessed in relation to previously published data for the Spanish population and were apparently consistent with our findings.

#### 3.2.4. Psychosocial Variables

The results concerning loneliness and the assessment of social isolation were similar (c.a. 12%). This percentage was double among interviewees living alone. The mean resilience value was close to the higher end of the intermediate interval of the scale (16.7 from a possible score of 17), although low resilience scores were recorded for 11% of the participants. In addition, the GDS-5 scale showed that 46 participants were at risk of depression while the PHQ-4 detected 40% at-risk cases of psychological distress.

#### 3.2.5. Health Problems

Hypertension, hypercholesterolemia, and diabetes were the most prevalent diseases in this population and, of note, 75% of the participants had at least one of these medical problems. Since each of these diseases constitute individual risk factors for cardiovascular events, the calculated risk of cardiovascular disease for the population fell into the high-risk category, while many of the participants were at a very high risk of cardiovascular disease. Only 31 respondents said they were active smokers.

#### 3.2.6. Lifestyle

Concerning the lifestyle of the population, 70% were overweight although their adherence to the Mediterranean diet pattern and physical activity levels were adequate. The mean cognitive reserve values detected were intermediate, while sleep problems were recorded in less than 20% of the patients.

#### 3.2.7. Healthy Aging

According to the Rowe and Kahn criteria, the calculated prevalence of HA was 6.6%. Considering each component of the Rowe and Kahn criteria separately, 165 (77.5%) had been diagnosed with a major illness, 48 (22.5%) were dependent, 52 (24.4%) were cognitively impaired, 50 (23.5%) were physically inactive, and 175 (82.2%) were obese or hypertense. Of the 200 patients who were not aging healthily, 46 (21.1%) met only one of these criteria, and from among these individuals, 25 had been diagnosed with a major illness and 13 were hypertensive.

### 3.3. Data Analysis

#### 3.3.1. Correlations between Variables

The Shapiro–Wilk normality test determined the use of a nonparametric correlation test. Results are shown with the false discovery rate (a *p*-value adjusted according to the number of tests performed) in Table S1. The minimum false discovery rate was 0.01711 for the LSNS and so we could not assume that the numerical variables were normally distributed. Thus, parametric tests were not appropriate and Spearman’s rho statistic was estimated as a rank measure of association. Following these criteria, pair-wise correlation tests were carried out, as shown in Table S2.

**Figure 1:**
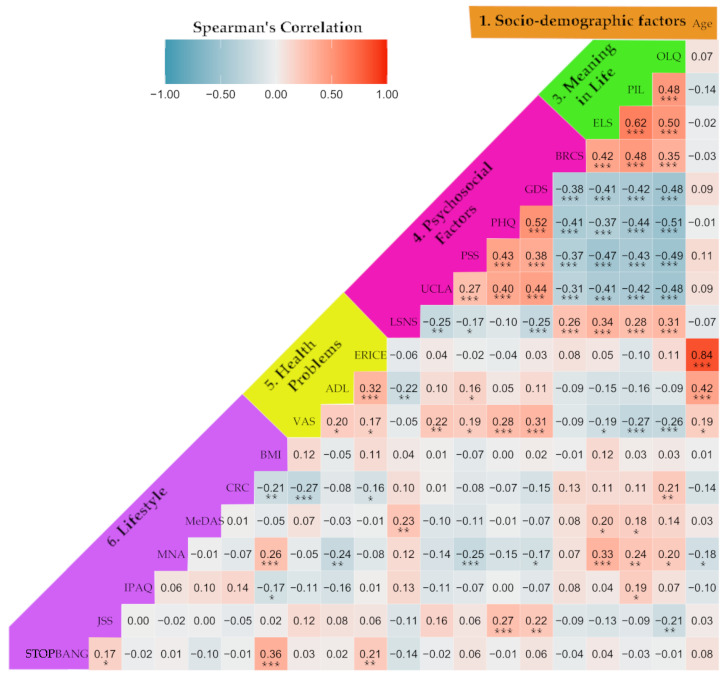
Correlogram of all the numerical variables. The Spearman’s rho estimation and significance levels are shown for each pair of variables. *: *p*-value < 0.05; **: *p*-value < 0.01; ***: *p*-value < 0.001.

Figure 1 shows a correlogram based on the Spearman’s rho values we obtained in this work. To aid our understanding, we grouped the variables following the classification provided in the previous tables. The intensity of the red and blue colors represents the degree of positive or negative correlation for the corresponding pair. Hence, we found multiple significant associations among the collected variables. For instance, the strongest correlations were 0.84 between age and ERICE and −0.51 between the Orientation to Life Questionnaire (OLQ) and Patient Health Questionnaire (PHQ). Furthermore, health problems such as chronic pain (measured on a visual analogue scale), nutrition through the Mini Nutritional Assessment (MNA), and sleep quality through the Jenkins Sleep Scale (JSS) were correlated with psychosocial variables and MiL domains. Psychosocial factors and MiL constructs were not significantly correlated according to age, although they were all interlinked.

#### 3.3.2. Correlations between Variables and Cognitive Impairment

Table 4 summarizes the variables that were significantly associated with CI. The results are shown following the same tabulation order and structure as Table 3. Population screening found 52 (24.4%) interviewees at risk of developing CI (the CI risk group). Concerning the sociodemographic factors, two non-modifiable variables were associated with CI: (i) age, which was significantly higher in the CI risk group (78.9 ± 7.7 vs. 71.0 ± 11.5) and (ii) marital status, with being married showing a protective effect compared to other marital statuses.

Regarding the CI section of the DeCo-B, the CI risk is significantly higher among the individuals reporting subjective memory complaints with a significance level lower than 0.1. SPMSQ test accounted for the higher percentage of positive results when screening cognition problems (13.1%). In terms of MiL, the average PiL test score was significantly higher for the no CI risk group (30.0 ± 5.4 vs. 28.2 ± 5.9) while the average score for the EwL test was significantly higher for the CI risk group (72.3 ± 13.1 vs. 69.5 ± 9.9).

In the psychosocial factors section, a higher score for resilience was associated with the no CI risk group (16.5 ± 3.0 vs. 15.5 ± 3.1) while hypertension, hypercholesterolemia, or diabetes were the health problems most frequently linked to a higher CI risk. Likewise, the ERICE score was significantly higher in the CI risk group (36.9 ± 15.9 vs. 26.0 ± 17.1) while patients reporting a higher degree of chronic pain were mostly in the CI risk group (4.2 ± 2.7 vs. 3.2 ± 2.8).

Regarding the variables included in the lifestyle section, both BMI and the mean *STOP–Bang Questionnaire* score were significantly higher in the CI risk group (28.3 ± 3.8 vs. 26.8 ± 4.1 and 3.5 ± 1.3 vs. 3.0 ± 1.5, respectively) but there was an inverse association for cognitive reserve in the CI risk group patients (8.2 ± 5.1 vs. 11.0 ± 5.0).

Based on the results shown in Table 4, we considered PiL, EwL, resilience, and chronic pain as the most important variables in CI risk assessment. We constructed four multivariate logistical regression models, adjusted for three age categories, (Table 5) to estimate the influence of age on PiL, EwL, resilience, and chronic pain associations with CI risk.

**Figure 2:**
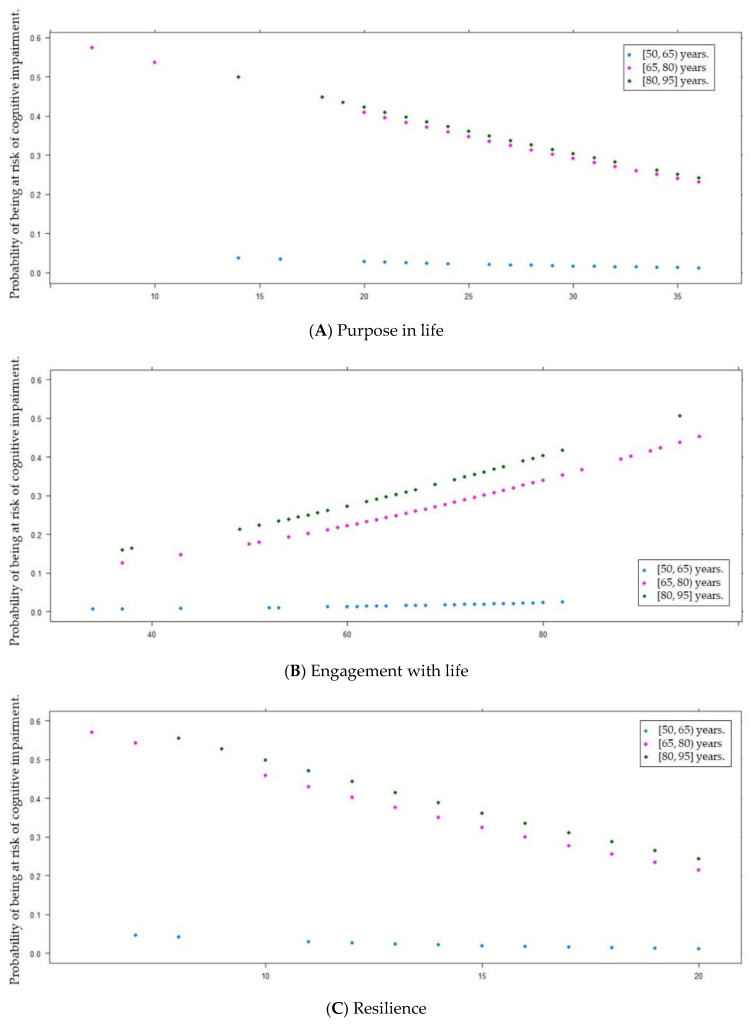

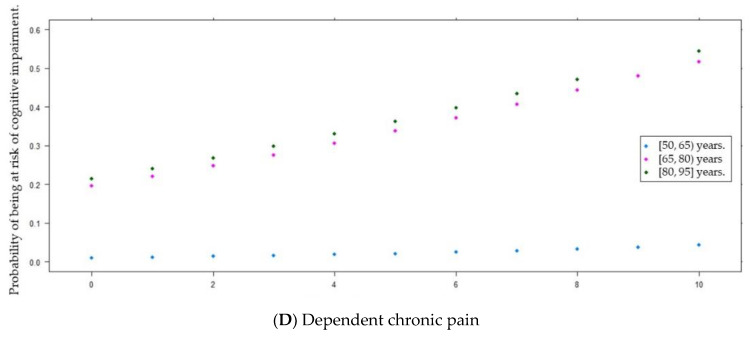
Estimated probability of being at risk of cognitive impairment based on (**A**) purpose in life; (**B**) engagement with life; (**C**) resilience; or (**D**) dependent chronic pain, adjusted by age groups.

The estimated CI risk probability was significantly lower in the younger category than in the other clusters. Although these two groups did not show differences in the CI risk probability, in both cases this probability decreased as PiL and resilience values increased (from 0.6 and 0.5 to 0.25, respectively). In turn, this probability increased to reach values close to 0.5 as the EwL and chronic pain values increased (see Figure 2).

## 4. Discussion

Detailed research analyzing the relationship between risk and protective factors affecting CI and HA in the Spanish population is lacking. Given that dementia is still incurable, early detection together with preventive actions seem to be the most advisable strategies for its management. The multifactorial nature of the disease calls for a holistic approach. Moreover, the psychosocial contribution seems to play an important role in the onset and outcome of the process. In this current work, we designed a booklet, the DeCo-B, comprising individually validated tools suitable for face-to-face population screening interviews. This allows the assessment of a wide range of variables encompassing dietary habits and social constraints in addition to well-being and satisfaction with life. To the best of our knowledge, no alternative tool for comprehensively evaluating CI risk factors is currently available.

The DeCo-B comprises more than 20 validated tools that allow us to characterize over 30 variables. Although no specific evaluation method was used to this end, the patients’ perceptions of the interview process seemed to be satisfactory, and the interview durations were also appropriate for research purposes. Nonetheless, optimization of the content and length will be one of our goals in developing the DeCo-B towards routine clinical application. Of note, the study of pharmacotherapy and pathologies is a time-consuming task in polymedicated and pluripathological participants which considerably increased the time required per patient because the researchers often had to perform additional work for data processing and analysis. However, the information extracted from these data may be very valuable because some drugs can influence the onset of CI in two different ways by acting (i) as a protective factor, as in the case of angiotensin II receptor antagonists [85] or (ii), as a RF, such as benzodiazepines and anticholinergic drugs [86,87]. Thus, this detailed evaluation is a critical step towards reducing the influence of these well-known, modifiable RFs, as well as towards detecting drug-related problems and preventing negative outcomes related to medicines.

Health professionals have very limited available time and so interventions driven by community pharmacists may be a convenient alternative for patient screening, especially in small populations with limited healthcare resources. In fact, close collaboration between community pharmacists, general practitioners, and neurologists increases the early detection of undiagnosed dementias, allowing better clinical follow-up of patients [27]. Given that the DeCo-B reflects a global overview of patient cognitive risk and status, it might be easily adapted for use as an initial screening method applicable in different risk populations (e.g., differing institutions, age groups, companies, etc.) or for pathologies. Thus, this strategy could form the basis of a convenient procedure not only allowing rapid participant screening, but also prioritizing the most important RFs that should be addressed on a case-by-case basis.

No extensive work is available on the prevalence of CI in the Spanish population apart from a recent study carried out with individuals aged over 65 visiting primary care sentinel physicians; these patients were representative of the overall population in five Spanish regions in terms of age, sex, and their place of residence. The prevalence of CI in this latter study varied between 26.6% and 11.7% [88], in line with the prevalence calculated for our sample (24.4%), which was also consistent with the figure for subjective memory complaints (27.5%) described in previous studies conducted in a Spanish population [27]. Although this type of memory complaint was not considered as an inclusion criterion in this current study, it was assessed in the CI section of the DeCo-B. The mean age of our participants was higher than in other studies (72.8 vs. 69.5 years) because we included patients aged up to 95 years while other studies limited this criterion to 80 years [27]. The associations between age and CI as well as subjective memory complaints and CI are well-established and were also noted in our results (see Table 4). Thus, it appears reasonable that the prevalence found for both populations was similar despite the individual contribution of each RF.

Furthermore, when we analyzed the links between CI and each assessed variable, we found at least one significant association in each section of the DeCo-B. Considering that this was a pilot study, we believe it would be interesting to further investigate all the variables that had a *p*-value < 0.1 (see Table 4).

Among the variables included in the sociodemographic section of the DeCo-B, being married seemed to play a protective role against the development of CI. Thus, compared to married individuals, widowers and singles had a 20% and 42% higher risk, respectively, of suffering from cognitive alterations [89]. In addition, married people were more likely to have healthy lifestyles, higher social support, and a wider social network, including both friends and family [90].

As far as the MiL section was concerned, our data were also consistent with the academic literature we reviewed. Considering each domain separately, the mean values measured in our study were only slightly different than those already reported for the Spanish population (for example, the PiL in our study was 29.3 vs. 29.0 and 30.3 in the literature). Moreover, these differences could again be discussed in terms of the mean age difference (72.8 ± 11.2 years in our sample vs. 54.3 ± 7.2 and 52.0 ± 7.1 in the literature). The interview design (in person vs. online) could have also been related to the differences in assessment and thus, in the measured values [26,43]. PiL is a domain that is protective against cognitive alterations and decline. Higher PiL values were associated with the presence of fewer deleterious effects of EA on cognitive function in older age [91] and increased PiL was also significantly related with dementia diagnoses and mortality at later stages in life [92]. Thus, several authors have proposed interventions focusing on positive psychology [93] designed to take advantage of the modifiable nature of PiL by increasing its values [94]. In fact, increasing PiL has positive effects on perceived memory loss and cognitive function both in the prodromic [46] and also the later phases of dementia [22].

PiL has also been significantly associated with resilience [45] and similarly, we observed a correlation between this factor and higher PiL (*p* < 0.01). Moreover, higher resilience values were associated with a lower CI risk in our sample. Resilience has been described as the ability to successfully cope with stress and difficult situations [95]. Recent research has shown that a lower ability to cope with stress may be an early sign of accumulating tau protein, a characteristic marker for AD, while a stronger coping ability may limit the negative effects of stress on tau deposition [96].

Although no further associations between CI and other psychosocial variables were found, the influence of social isolation and loneliness on CI has been established [6]. Moreover, we also observed some of the pairwise associations between risk and protective factors already described in the literature; for example, loneliness has been associated with MiL [43]. According to our data, all the psychosocial and MiL variables were positively or negatively correlated with CI and an imbalance in these variables could indicate the development of CI. Thus, more thorough studies will be required to analyze the degree to which each factor contributes to this risk and to obtain a broader picture of the relationship between risk and protective variables.

According to the academic literature [6], 40% of the modifiable dementia risk can be explained by RFs such as hypertension, diabetes, depression, and social isolation, among others. Our results showed a higher prevalence of CI in patients suffering from these pathologies (*p* < 0.05 for hypertension and diabetes; *p* < 0.1 for hypercholesterolemia). Indeed, a higher cardiovascular risk in middle age increases the risk of cognitive decline [97,98,99]. In the same line, higher ERICE scores in our work were associated with positive CI screening results. The same was true for chronic pain, an unspecific symptom which can be associated with numerous pathologies and is known to correlate with cognitive decline [100]. In addition, the proposed molecular mechanisms of chronic pain suggest that it may also accelerate the pathogenesis of AD [101]. Therefore, a more detailed analysis considering confusing variables such as age would be advisable.

Among lifestyle variables significantly associated with a lower risk of CI in this work, high cognitive reserve particularly stood out. In fact, the attainment of higher lifelong education levels is thought to reduce the risk of dementia. It has also been proposed that increased cognitive reserve could explain why some individuals maintain cognitive abilities despite impaired brain health due to physiological (aging) or pathological (diseases) [6,102]. Interestingly, CI is a recognized clinical consequence of obstructive sleep apnea; it is thought that this association is based on resulting hypoxic conditions and oxidative stress processes occurring in brain tissues, as well as hemodynamic changes and cardiovascular comorbidities. All of the above makes obstructive sleep apnea a potential modifiable target for AD prevention [103]. We also found a significant association between CI and low participation in group community activities. We believe that this may be related to the sharp decrease in social interactions of this kind during the COVID-19 lockdown.

According to our data from the DeCo-B, the prevalence of HA in our population was close to 6.6%. Rowe and Kahn’s model provides an initial quick estimation of HA and its comparison between populations, although some authors consider it to be excessively restrictive and several modifications for calculating the prevalence of HA have been proposed. For example, McLaughlin [104] and Rodriguez-Laso [105] conceptualize and estimate HA prevalence at four different levels: (i) Rowe and Kahn’s definition; (ii) level 1: the same as (i) but excluding the freedom from RF criterion (obesity and self-referred hypertension) and using a threshold for CI; (iii) level 2: the same as (ii) but including only limitations in the performance of daily activities as disease criterion; and (iv) level 3: the same as (ii) but excluding disease criterion entirely. Thus, the calculated HA prevalence for our population according to these levels was 16.4% (*n* = 35) for level 1, 30.5% (*n* = 65) for level 2, and 62% (*n* = 113) for level 3. Inclusion of concepts such as the degree of chronic disease control and recovery from serious illnesses may also be suitable for a more current and accurate conceptualization of HA. However, once a less restrictive definition is agreed, the lack of psychosocial variables in the prevalence calculation may lead to estimation errors. Of note, in Rowe and Khan’s model, EwL is defined as participation in social activities and doing paid work and does not refer to the MiL domain. However, managing physical, psychological, and cognitive health may be a cornerstone for modulating the most decisive RF for developing cognitive decline: age.

We considered Rowe and Kahn’s original model so that we could compare our findings with previously published prevalences, which in our case, were higher (6.6%) than those already published for Spanish samples (4.5% and 3.1%, respectively) [105,106]. When discussing existing data, the contribution of CI and physical function assessment to HA prevalence calculations should be considered because the use of distinct evaluation tools for these variables could cause differences in these factors. However, the main differences can be explained by considering the fact that our mean sample age (which included participants aged over 50 years) was remarkably lower than other cohorts described in the literature. In fact, if we calculate the prevalence of HA in our sample only for participants aged 65 and older, the result drops to 3.6%, in concordance with published data. Of note, the prevalence of HA reported for European countries was significantly higher (mean of 8.5%; France: 8.4%, Italy: 5.3%, and Denmark: 21.1%) although the life expectancy of the Spanish population stands out as one of the highest in the world. In summary, going beyond the theoretical model and crude HA prevalence percentages, it would be reasonable to think that protective factors may influence aging, despite its complexity as a process. Thus, this would lead us to believe that psychosocial variables such as resilience, belongingness, and positive mental health, as well as MiL, might somehow contribute to the overall HA process.

The main limitations of this investigation are the length of the interview, the number of participants, and interviewer bias. As far as strengths are concerned, the DeCo-B comprises 23 validated tools suitable for face-to-face interview and assesses 31 different variables. Moreover, this questionnaire can easily be adapted to different situations or risk populations. Regarding novel aspects, to the best of our knowledge, there is no alternative tool for comprehensively evaluating CI risk factors currently available, especially psychosocial variables and MIL. Finally, it is worth mentioning that this is a cross-sectional investigation. To better understand the causal nature of observed associations, longitudinal research is needed.

## 5. Conclusions

The joint assessment of the influence of psychosocial, clinical, and lifestyle-related variables on CI through a compilation of validated tests provides a useful pool of information for analyzing the course of cognitive alterations. The numerous, complex interrelationships between risk and protective factors confirm the need to expand efforts to characterize the most influential variables. In this context, our results suggest that further research on psychosocial factors, such as resilience and MiL domains, may provide opportunities for a better understanding of cognitive decline and HA. Development of multifactorial strategies that include psychosocial variables for promoting cognitive health constitute a promising field of research. In this respect, novel areas, such as social isolation and cognitive reserve, should also be the focus of efforts to advance the CI knowledge.

## Figures and Tables

**Table 1:** Inclusion and exclusion criteria for meaning in life and psychosocial factors used for the bibliographic literature review.

Inclusion Criteria	Exclusion Criteria
Validated scales	Non-validated or ad hoc tools
Scales translated into Spanish or tested in the Spanish population	Non-community dwelling population Population aged under 50 years
Suitable for a face-to-face interview	Cognitive impairment or Alzheimer’s disease at the baseline
Administration time and/or length ensures a suitable interview length (maximum of 10 min)	Literature related to COVID-19 disease

**Table 3:** The structure of the DeCo-B and pilot study results.

Structure of the Data Collection Booklet			Pilot Study Results	
Ref.	No. of Items	Variable, Validated Test/Criteria, and Categories	*N* (%) or Mean ± SD	Time (min)
		**1. Sociodemographic Factors**		
	4	Sex		1
		Women	131 (61.2)	
		Men	81 (37.9)	
		Age	72.8 ± 11.2	
		[50, 65)	57 (27.1)	
		[65, 80)	85 (40.5)	
		[80, 95]	68 (32.4)	
		Marital status		
		Married	139 (65.6)	
		Widow	57 (26.6)	
		Separated/divorced	6 (2.8)	
		Single	10 (4.7)	
		Family history of dementia		
		Yes	34 (16.0)	
		No	179 (84.0)	
		**2. Cognitive Impairment**		
	1	Subjective memory complaint	79 (37.1)	<1
[30]	8	Memory Impairment Screen (MIS) [0, 8]	6.8 ± 2.0	2
		CI risk [0, 4]	21 (9.9)	
		No CI risk (4, 8]	191 (90.1)	
[32]	10	Short Portable Mental State Questionnaire (SPMSQ) [0–10]	1.1 ± 1.3	3
		CI risk [3–4, 10]	28 (13.2)	
		No CI risk [0, 3–4)	185 (87.0)	
[31]	1	Semantic Verbal Fluency (SVF) [0, +∞)	17.5 ± 7.1	1
		CI risk [0, 10]	16 (7.5)	
		No CI risk [10, +∞)	196 (92.5)	
		**3. Meaning in Life**		
[66]	13	Sense of coherence		8
		Orientation to Life Questionnaire (OLQ-13) [13, 91]	69.1 ± 11.9	
[67]	6	Purpose in life		4
		PiL subscale of Ryff’s Well-Being Scale (PiL) [6, 36]	29.6 ± 5.6	
[68]	16	Engagement with life		8
		Engaged Living Scale (ELS) [16, 80]	70.1 ± 10.8	
		**4. Psychosocial Factors**		
[69]	4	Resilience		2
		Brief Resilient Coping Scale (BRCS) [4, 20]	16.7 ± 3.0	
		Low [4, 13)	24 (11.3)	
		Intermediate [13, 17]	104 (48.8)	
		High (17, 20]	85 (39.9)	
[70]	5	Depression		1
		Geriatric Depression Scale (GDS-5) [0, 5]	0.8 ± 1.3	
		Risk of depression [2, 5]	46 (21.6)	
		No risk of depression [0, 2)	167 (78.4)	
[71]	4	Psychological distress		2
		Patient Health Questionnaire-4 (PHQ-4) [0–12]	2.6 ± 2.6	
		None [0, 2]	126 (59.2)	
		Mild [3, 5]	57 (26.8)	
		Moderate [6, 8]	22 (10.3)	
		Severe [9, 12]	8 (3.8)	
[72]	4	Stress		2
		Perceived Stress Scale (PSS-4) [0, 16]	3.8 ± 3.1	
		Perceived stress (5.4, 16]	59 (27.7)	
		No perceived stress [0, 5.4)	154 (72.3)	
[73]	3	Loneliness		1
		UCLA-3 [3, 9]	3.9 ± 1.5	
		Loneliness [6, 9]	27 (12.7)	
		No loneliness [3, 6)	186 (87.3)	
[74]	6	Social isolation		5
		Lubben Social Network Scale (LSNS-6) [0, 30]	18.8 ± 5.0	
		Social isolation [0, 12]	24 (11.3)	
		No social isolation (12, 30]	189 (88.7)	
		**5. Health Problems**		
[75]	1	Hypertension		5
		Yes [systolic BP > 140 mmHg–diastolic BP > 90 mmHg]	126 (60.0)	
		No [systolic BP ≤ 140 mmHg–diastolic BP ≤ 90 mmHg]	84 (40.0)	
[76]	1	Hypercholesterolemia		
		Yes [C_T_ > 200 mg/dL–C_LDL_ > 100 mg/dL–C_HDL_ ≤ 35–40 mg/dL]	94 (44.8)	
		No [C_T_ ≤ 200 mg/dL–C_LDL_ ≤ 100 mg/dL–C_HDL_ > 35–40 mg/dL]	116 (55.2)	
[77]	1	Diabetes		
		Yes [blood glucose > 126 mg/dL–HbA1c > 6.5%]	50 (23.5)	
		No [blood glucose ≤ 126 mg/dL–HbA1c < 6.5%]	163 (76.5)	
	1	Smoking habit		<1
		Non-smoker	117 (54.9)	
		Former smoker	53 (24.9)	
		Passive smoker	12 (5.6)	
		Smoker	31 (14.6)	
[39]	7	Risk of cardiovascular disease		1
		ERICE Scale [1, 84]	28.5 ± 17.4	
		Low [1, 5)	1 (0.5)	
		Mild [5, 9]	37 (17.4)	
		Moderate [10, 14]	18 (8.5)	
		Moderate–high [15, 19]	17 (8.0)	
		High [20, 29]	42 (19.7)	
		Very high [30, 84]	92 (42.2)	
[40]	11	Dependency		1
		Independent for P-ADL and I-ADL [0, 11]	0.8 ± 2.0	
		Dependent [1, 11]	48 (22.5)	
	1	Hearing loss		<1
		Yes [self-perceived]	95 (44.6)	
		No	118 (55.4)	
	1	Chronic pain		<1
		Visual analogue scale (VAS) [0–10]	3.4 ± 2.8	
		No pain [0]	54 (25.4)	
		Mild [1, 3]	57 (26.8)	
		Moderate [4, 6]	65 (30.5)	
		Severe [7, 8]	23 (10.8)	
		Excruciating [9, 10]	9 (4.2)	
		**6. Lifestyle**		
[78]	2	Anthropometry		<1
		WHO stepwise method		
		BMI (kg/m^2^)	27.2 ± 4.1	
		Normal weight [18.5, 25)	61 (28.6)	
		Overweight [25, 30)	96 (46.6)	
		Obese [30, +∞)	49 (23.8)	
	1	Retired		<1
		Yes	145 (68.7)	
		No	66 (31.3)	
[79]	8	Cognitive reserve		2
		Cognitive Reserve Questionnaire (CRC) [0, 25]	10.3 ± 5.1	
		Low [0, 6]	55 (25.9)	
		Intermediate–low [7, 9]	46 (21.7)	
		Intermediate–high [10, 14]	65 (30.7)	
		High [15, 25]	46 (21.7)	
		Nutrition		
[80]	14	Mediterranean Diet Adherence Score (MeDAS) [0, 14]	9.2 ± 2.1	3
		Low [0, 6]	20 (9.4)	
		Intermediate [7, 9]	99 (46.7)	
		High [10, 14]	93 (43.9)	
[81]	6	Mini Nutritional Assessment (MNA) [0–14]	12.2 ± 1.9	2
		Risk of malnutrition [0, 12)	74 (34.7)	
		Normal nutrition [12, 14]	139 (65.0)	
[82]	7	Physical activity		5
		International Physical Activity Questionnaire (IPAQ)		
		Low	50 (23.5)	
		Moderate	95 (44.6)	
		High	68 (31.9)	
		Sleep		
[83]	4	Jenkins Sleep Scale (JSS) [0, 20]	7.38 ± 5.0	2
		Sleep disorder [12, 20]	41 (19.3)	
		No sleep disorder [0, 12)	172 (80.8)	
[84]	8	STOP-Bang Questionnaire [0, 8]	3.1 ± 1.4	2
		Low [0, 2]	81 (38.0)	
		Intermediate [3–4]	97 (45.5)	
		High [5, 8]	35 (16.4)	
Total	159	No. of variables = 31; No. of tests = 23		69

Numerical results are described as means and standard deviations (mean ± SD) while qualitative results are described with sample sizes and percentages (*n* (%)). Abbreviations: BP: blood pressure; C_T_: total cholesterol; C_LDL_: low-density lipoprotein cholesterol; C_HDL_: high-density lipoprotein cholesterol; HbA1c: hemoglobin A1c; P-ADL: personal activities of daily living; and I-ADL: instrumental activities of daily living.

**Table 4:** Variables associated with cognitive impairment.

Variable	Totals *n* (%) 213 (100)	Risk CI *n* (%) 52 (24.4)	No Risk CI *n* (%) 161 (75.6)	*p*-Value
**1. Sociodemographic Factors**				
Age				
($\overset{¯}{x}$ ± s)	72.8 ± 11.2	78.9 ± 7.7	71.0 ± 11.5	<0.001 ^a^ ***
[50, 65)	57 (27.1)	1 (2.0)	56 (35.0)	<0.001 ^b^ ***
[65, 80)	85 (40.5)	25 (51.0)	60 (37.0)	
[80, 95]	68 (32.4)	23 (47.0)	45 (28.0)	
Marital status				
Married	139 (65.6)	26 (51.0)	113 (70.2)	0.013 ^b^ *
Widow	57 (26.9)	22 (43.1)	35 (21.7)	
Separated/Divorced	6 (2.8)	0 (0.0)	6 (3.7)	
Single	10 (4.7)	3 (5.9)	7 (4.3)	
**2. Cognitive Impairment**				
Subjective memory complaint				
Yes	79 (37.1)	25 (48.1) 27 (51.9)	54 (33.5) 107 (66.5)	0.070 ^c^
No	134 (62.9)			
Memory Impairment Screen (MIS) [0, 8]				
($\overset{¯}{x}$ ± s)	6.8 ± 1.9	4.9 ± 2.7	7.4 ± 1.1	<0.001 ^d^ ***
CI risk [0, 4]	21 (9.9)	21 (40.4)	0 (0.0)	<0.001 ^b^ ***
No CI risk (4, 8]	191 (90.1)	31 (59.6)	160 (100.0)	
Short Portable Mental State Questionnaire (SPMSQ) [0, 10]				
($\overset{¯}{x}$ ± s)	1.1 ± 1.3	2.4 ± 1.6	0.7 ± 0.9	<0.001 ^d^ ***
CI risk [3–4, 10) No CI risk [0, 3–4)	28 (13.1)	28 (53.8)	0 (0.0)	<0.001 ^b^ ***
	185 (86.9)	24 (46.2)	161 (100.0)	
Semantic Verbal Fluency (SVF) [0, +∞) ($\overset{¯}{x}$ ± s)	17.4 ± 7.1	13.5 ± 5.1	18.7 ± 7.2	<0.001 ^d^ ***
CI risk [0, 10] No CI risk [10, +∞)	16 (7.5)	16 (30.8)	0 (0.0)	<0.001 ^b^ ***
	196 (92.5)	36 (69.2)	160 (100.0)	
**3. Meaning in Life**				
Purpose in life (PiL) [6, 36] ($\overset{¯}{x}$ ± s)	29.6 ± 5.6	28.2 ± 5.9	30.0 ± 5.4	0.020 ^d^ *
Engagement with life (EwL) [16, 80] ($\overset{¯}{x}$ ± s)	70.1 ± 10.8	72.3 ± 13.1	69.5 ± 9.9	0.016 ^d^ *
**4. Psychosocial Factors**				
Brief Resilient Coping Scale (BRCS) [4, 20] ($\overset{¯}{x}$ ± s)	16.3 ± 3.0	15.5 ± 3.1	16.5 ± 3.0	0.018 ^d^ *
Low [4, 13) Intermediate [13, 17] High (17, 20]	24 (11.3)	10 (19.2)	14 (8.7)	0.075 ^c^ †
	104 (48.8)	26 (50.0)	78 (48.4)	
	85 (39.9)	16 (30.8)	69 (42.9)	
**5. Health Problems**				
Hypertension				
Yes [systolic BP > 140 mmHg or diastolic BP > 90 mmHg] No [systolic BP ≤ 140 mmHg and diastolic BP ≤ 90 mmHg]	126 (60.0)	38 (76.0)	88 (55.0)	0.008 ^c^ **
	84 (40.0)	12 (24.0)	72 (45.0)	
Hypercholesterolemia				
Yes [Total > 200 mg/dL or LDL >100 mg/dL or HDL ≤ 35–40 mg/dL] No [Total ≤ 200 mg/dL or LDL ≤ 100 mg/dL or HDL > 35–40 mg/dL]	94 (44.8)	29 (55.8)	65 (41.1)	0.078 ^c^ †
	116 (55.2)	23 (44.2)	93 (58.9)	
Diabetes				
Yes [Blood glucose > 126 mg/dL or HbA1c > 6.5%] No [Blood glucose ≤ 126 mg/dL and HbA1c ≤ 6.5%]	50 (23.5)	18 (34.6)	32 (19.9)	0.038 ^c^ *
	163 (76.5)	34 (65.4)	129 (80.1)	
ERICE Scale [1, 84] ($\overset{¯}{x}$ ± s)	28.4 ± 17.4	36.9 ± 15.9	26.0 ± 17.1	<0.001 ^d^ ***
Low [1, 5)	1 (0.5)	0 (0.0)	1 (0.6)	0.002 ^b^ **
Mild [5, 9]	37 (17.9)	3 (6.4)	34 (21.2)	
Moderate [10, 14]	18 (8.7)	1 (2.1)	17 (10.6)	
Moderate–high [15, 19]	17 (8.2)	2 (4.3)	15 (9.4)	
High [20, 29]	42 (20.3)	8 (17.0)	34 (21.2)	
Very high [30, 80]	92 (44.4)	33 (70.2)	59 (36.9)	
Dependent chronic pain [0, 10] ($\overset{¯}{x}$ ± s)	3.4 ± 2.8	4.2 ± 2.7	3.2 ± 2.8	0.013 ^d^ *
No pain [0]	54 (26.0)	5 (10.0)	49 (31.0)	0.018 ^b^ *
Mild [1, 3]	57 (27.4)	17 (34.0)	40 (25.3)	
Moderate [4, 6]	65 (31.2)	19 (38.0)	46 (29.1)	
Severe [7, 8]	23 (11.1)	5 (10.0)	18 (11.4)	
Unbearable [9, 10]	9 (4.3)	4 (8.0)	5 (3.2)	
**6. Lifestyle**				
BMI ($\overset{¯}{x}$ ± s)	27.2 ± 4.1	28.3 ± 3.8	26.8 ± 4.1	0.017 ^a^ *
Normal weight [18.5, 25)	61 (29.6)	8 (16.3)	53 (33.9)	0.046 ^c^ *
Overweight [25, 30)	96 (46.6)	26 (51.3)	70 (44.6)	
Obese [30, +∞)	49 (23.8)	15 (30.6)	34 (21.7)	
Retired				
Yes No	145 (68.7)	48 (94.1)	97 (60.6)	<0.001 ^b^ ***
	66 (31.3)	3 (5.9)	63 (39.4)	
Cognitive Reserve Questionnaire (CRC) [0, 25] ($\overset{¯}{x}$ ± s)	10.3 ± 5.1	8.2 ± 5.1	11.0 ± 5.0	<0.001 ^d^ ***
Low [0, 6]	55 (25.9)	22 (43.1)	33 (20.5)	0.010 ^b^ *
Intermediate–low [7, 9]	46 (21.7)	11 (21.6)	35 (21.7)	
Intermediate–high [10, 14]	65 (30.7)	12 (23.5)	53 (32.9)	
High [15, 25]	46 (21.7)	6 (11.8)	40 (24.8)	
STOP–Bang Questionnaire [0, 8] ($\overset{¯}{x}$ ± s)	3.1 ± 1.4	3.5 ± 1.3	3.0 ± 1.5	0.005 ^d^ **
Low [0, 2]	81 (38.0)	13 (25.0)	68 (42.2)	0.075 ^b^ †
Intermediate [3, 4]	97 (45.5)	28 (53.8)	69 (42.9)	
High [5, 8]	35 (16.4)	11 (21.2)	24 (14.9)	

Variables associated with cognitive impairment. ^a^: *t*-test for comparison of two independent means (one-sided); ^b^: Fisher’s exact test; ^c^: Chi-squared test; ^d^: Wilcoxon test for two independent means (one-sided); †: *p*-value < 0.1; *: *p*-value < 0.05; **: *p*-value < 0.01; ***: *p*-value < 0.001.

**Table 5:** Logistic regression models for the risk of cognitive decline adjusted for categorized age and purpose in life (model A), engagement with life (model B), resilience (model C), or dependent chronic pain (model D).

Variable	β_i_	*SD*	Wald	d.f.	*p*-Value	Exp(β_i_)	95% CI	
							UL	LL
Model A								
Intercept	−2.51	1.34	−1.87	1	<0.1 †	0.08	0.00	0.86
Age (50, 65) (65, 80) (80, 95)	- 3.17 3.23	- 1.04 1.04	- 3.05 3.09	- 1	- <0.01 ** <0.01 **	- 23.81 25.20	- 4.77 4.94	- 433.32 461.20
Purpose in life	−0.05	0.03	−1.66	1	<0.1 †	0.95	0.89	1.01
Model B								
Intercept	−6.10	1.60	−3.80	1	<0.001 ***	0.00	0.00	0.04
Age (50, 65) (65, 80) (80, 95)	- 3.08 3.35	- 1.04 1.04	- 2.97 3.20	- 1	- <0.01 ** <0.01 **	- 21.71 28.52	- 4.35 5.59	- 394.88 522.08
Engagement with life	0.03	0.02	1.69	1	<0.1 †	1.03	1.00	1.07
Model C								
Intercept	−2.24	1.32	−1.69	1	<0.1 †	0.11	0.00	1.09
Age (50, 65) (65, 80) (80, 95)	- 3.20 3.37	- 1.04 1.04	- 3.08 3.22	- 1	- <0.01 ** <0.01 **	- 24.59 28.97	- 4.91 5.72	- 447.94 529.72
Resilience	−0.11	0.07	−2.01	1	<0.05 *	0.89	0.80	1.00
Model D								
Intercept	−4.57	1.05	−4.33	1	<0.001 ***	0.01	0.00	0.95
Age (50, 65) (65, 80) (80, 95)	- 3.16 3.27	- 1.04 1.05	- 3.03 3.13	- 1	- <0.01 ** <0.01 **	- 23.61 26.33	- 4.68 5.16	- 431.38 482.29
Dependent chronic pain	0.15	0.07	2.22	1	<0.05 *	1.16	1.02	1.32

βi: model coefficients; *SD*: standard deviation of the coefficients; d.f.: degrees of freedom; Exp(βi): odds ratio; UL: upper limit of the 95% confidence interval for the expected odds ratio; LL: lower limit of the 95% confidence interval for the expected odds ratio; †: *p*-value < 0.1; *: *p*-value < 0.05; **: *p*-value < 0.01; and ***: *p*-value < 0.001.

## Data Availability

The data used for this study are available upon request.

## Supplementary Materials

The following supporting information can be downloaded at: https://www.mdpi.com/article/10.3390/ijerph191912911/s1, Table S1. Results of Shapiro–Wilk test applied to the numerical variables. Table S2. Results of the test pairwise correlations applied to the numerical variables.
