# Supplementary material for: Addressing Psychosocial Factors in Cognitive Impairment Screening from a Holistic Perspective: The DeCo-Booklet Methodology Design and Pilot Study
Source: Int J Environ Res Public Health. 2022 Oct 9;19(19):12911. doi: 10.3390/ijerph191912911 (PMC9565987; doi:10.3390/ijerph191912911)
Supplement: Supplementary file 1 [file ijerph-19-12911-s001.zip › Table S1.pdf]

Table S1. Results of Shapiro-Wilk test applied to the numerical variables.

Variable	n	Statistic	p.Value	fdr
ADL	213	0.4752	1.081×10^{-24}	2.163×10^{-23} ***
GDS	213	0.6823	8.335×10^{-20}	8.335×10^{-19} ***
UCLA	213	0.7254	1.717×10^{-18}	1.144×10^{-17} ***
IPAQ	213	0.7406	5.393×10^{-18}	2.696×10^{-17} ***
PHQ	213	0.8609	5.116×10^{-13}	2.046×10^{-12} ***
MNA	213	0.8642	7.702×10^{-13}	2.567×10^{-12} ***
PIL	210	0.9051	2.625×10^{-10}	7.5×10^{-10} ***
BRCS	213	0.92	2.482×10^{-9}	6.206×10^{-9} ***
VAS	208	0.9191	2.925×10^{-9}	6.499×10^{-9} ***
PSS	213	0.9229	4.086×10^{-9}	8.171×10^{-9} ***
STOPBANG	213	0.9367	5.498×10^{-8}	9.997×10^{-8} ***
ERICE	207	0.9491	1.063×10^{-6}	1.771×10^{-6} ***
ELS	209	0.9502	1.219×10^{-6}	1.876×10^{-6} ***
JSS	213	0.9556	3.525×10^{-6}	5.035×10^{-6} ***
OLQ	211	0.9635	2.946×10^{-5}	3.928×10^{-5} ***
MeDAS	213	0.9681	9.58×10^{-5}	0.0001198 ***
Age	210	0.9701	0.0001938	0.000228 ***
CRC	212	0.9764	0.001259	0.001398 **
BMI	206	0.9834	0.01555	0.01637 *
LSNS	213	0.9841	0.01711	0.01711 *

n: sample size; fdr: false discovery rate. *: p-value < 0.05; **: p-value < 0.01; ***: p-value < 0.001.
