# Supplementary material for: Addressing Psychosocial Factors in Cognitive Impairment Screening from a Holistic Perspective: The DeCo-Booklet Methodology Design and Pilot Study
Source: Int J Environ Res Public Health. 2022 Oct 9;19(19):12911. doi: 10.3390/ijerph191912911 (PMC9565987; doi:10.3390/ijerph191912911)
Supplement: Supplementary file 1 [file ijerph-19-12911-s001.zip › Table S2.pdf]

Table S2. Results of the test pairwise correlations applied to the numerical variables.

Formula	n	Estimate	Statistic	p.Value	fdr
~Age+ERICE	207	0.8385	2.388×10^{5}	5.993×10^{-56}	1.139×10^{-53} ***
~PIL+ELS	209	0.6184	5.806×10^{5}	1.918×10^{-23}	1.822×10^{-21} ***
~GDS+PHQ	213	0.5154	7.805×10^{5}	7.533×10^{-16}	4.771×10^{-14} ***
~OLQ+PHQ	211	-0.5147	2.371×10^{6}	1.138×10^{-15}	5.407×10^{-14} ***
~OLQ+ELS	209	0.4969	7.655×10^{5}	1.98×10^{-14}	7.523×10^{-13} ***
~OLQ+PSS	211	-0.4933	2.338×10^{6}	2.427×10^{-14}	7.686×10^{-13} ***
~OLQ+PIL	210	0.4846	7.956×10^{5}	9.196×10^{-14}	2.496×10^{-12} ***
~OLQ+GDS	211	-0.4792	2.316×10^{6}	1.646×10^{-13}	3.909×10^{-12} ***
~OLQ+UCLA	211	-0.4755	2.31×10^{6}	2.65×10^{-13}	5.373×10^{-12} ***
~PIL+BRCS	210	0.476	8.087×10^{5}	2.828×10^{-13}	5.373×10^{-12} ***
~ELS+PSS	209	-0.4716	2.239×10^{6}	5.724×10^{-13}	9.887×10^{-12} ***
~GDS+UCLA	213	0.4419	8.989×10^{5}	1.357×10^{-11}	2.149×10^{-10} ***
~PIL+PHQ	210	-0.4418	2.225×10^{6}	1.904×10^{-11}	2.783×10^{-10} ***
~PHQ+PSS	213	0.4279	9.214×10^{5}	6.79×10^{-11}	8.715×10^{-10} ***
~PIL+PSS	210	-0.4306	2.208×10^{6}	6.88×10^{-11}	8.715×10^{-10} ***
~Age+ADL	210	0.4214	8.93×10^{5}	1.89×10^{-10}	2.245×10^{-9} ***
~PIL+GDS	210	-0.4185	2.189×10^{6}	2.587×10^{-10}	2.891×10^{-9} ***
~BRCS+PHQ	213	-0.4142	2.278×10^{6}	3.075×10^{-10}	3.12×10^{-9} ***
~PIL+UCLA	210	-0.4168	2.187×10^{6}	3.12×10^{-10}	3.12×10^{-9} ***
~ELS+BRCS	209	0.4164	8.88×10^{5}	3.604×10^{-10}	3.423×10^{-9} ***
~ELS+UCLA	209	-0.4059	2.139×10^{6}	1.075×10^{-9}	9.722×10^{-9} ***
~ELS+GDS	209	-0.4051	2.138×10^{6}	1.166×10^{-9}	1.007×10^{-8} ***
~PHQ+UCLA	213	0.3983	9.691×10^{5}	1.643×10^{-9}	1.357×10^{-8} ***
~BRCS+GDS	213	-0.3832	2.228×10^{6}	7.369×10^{-9}	5.834×10^{-8} ***
~GDS+PSS	213	0.3811	9.968×10^{5}	9.085×10^{-9}	6.904×10^{-8} ***

Formula	n	Estimate	Statistic	p.Value	fdr
~ELS+PHQ	209	-0.3735	2.09×10^{6}	2.554×10^{-8}	1.866×10^{-7} ***
~BRCS+PSS	213	-0.3691	2.205×10^{6}	2.812×10^{-8}	1.979×10^{-7} ***
~OLQ+BRCS	211	0.3537	1.012×10^{6}	1.298×10^{-7}	8.809×10^{-7} ***
~BMI+STOPBANG	206	0.3551	9.395×10^{5}	1.625×10^{-7}	1.065×10^{-6} ***
~ELS+LSNS	209	0.3351	1.012×10^{6}	7.077×10^{-7}	4.482×10^{-6} ***
~ELS+MNA	209	0.3317	1.017×10^{6}	9.26×10^{-7}	5.676×10^{-6} ***
~ERICE+ADL	207	0.3238	9.997×10^{5}	1.949×10^{-6}	1.157×10^{-5} ***
~BRCS+UCLA	213	-0.3109	2.111×10^{6}	3.744×10^{-6}	2.155×10^{-5} ***
~GDS+VAS	208	0.3135	1.03×10^{6}	3.996×10^{-6}	2.233×10^{-5} ***
~OLQ+LSNS	211	0.311	1.079×10^{6}	4.12×10^{-6}	2.237×10^{-5} ***
~PHQ+VAS	208	0.2831	1.075×10^{6}	3.416×10^{-5}	0.0001803 ***
~PIL+LSNS	210	0.2771	1.116×10^{6}	4.673×10^{-5}	0.00024 ***
~PSS+UCLA	213	0.2724	1.172×10^{6}	5.623×10^{-5}	0.0002812 ***
~PHQ+JSS	213	0.2665	1.181×10^{6}	8.238×10^{-5}	0.0004013 ***
~PIL+VAS	205	-0.2709	1.825×10^{6}	8.541×10^{-5}	0.0004042 ***
~VAS+CRC	208	-0.2687	1.903×10^{6}	8.723×10^{-5}	0.0004042 ***
~OLQ+VAS	206	-0.2622	1.839×10^{6}	0.0001408	0.0006369 ***
~BRCS+LSNS	213	0.256	1.198×10^{6}	0.000159	0.0007024 ***
~GDS+LSNS	213	-0.2549	2.021×10^{6}	0.0001693	0.000728 ***
~BMI+MNA	206	0.2588	1.08×10^{6}	0.0001724	0.000728 ***
~PSS+MNA	213	-0.2512	2.015×10^{6}	0.000212	0.0008755 ***
~UCLA+LSNS	213	-0.2472	2.009×10^{6}	0.0002689	0.001087 **
~ADL+MNA	213	-0.2438	2.003×10^{6}	0.0003296	0.001305 **
~PIL+MNA	210	0.2361	1.179×10^{6}	0.0005602	0.002172 **
~LSNS+MeDAS	213	0.2337	1.234×10^{6}	0.0005859	0.002226 **
~GDS+JSS	213	0.2204	1.256×10^{6}	0.001207	0.004495 **
~UCLA+VAS	208	0.221	1.168×10^{6}	0.001337	0.004886 **

Formula	n	Estimate	Statistic	p.Value	fdr
~LSNS+ADL	213	-0.2151	1.957×10^6	0.001593	0.005711 **
~BMI+CRC	206	-0.2134	1.768×10^6	0.002073	0.007293 **
~OLQ+JSS	211	-0.2105	1.895×10^6	0.002113	0.0073 **
~ERICE+STOPBANG	207	0.2082	1.17×10^6	0.00261	0.008854 **
~OLQ+CRC	210	0.2061	1.225×10^6	0.002683	0.008944 **
~ELS+MeDAS	209	0.2018	1.215×10^6	0.003396	0.01113 *
~OLQ+MNA	211	0.1989	1.254×10^6	0.003711	0.01195 *
~ADL+VAS	208	0.1955	1.207×10^6	0.004647	0.01471 *
~PIL+IPAQ	210	0.192	1.247×10^6	0.005241	0.01632 *
~PSS+VAS	208	0.1925	1.211×10^6	0.00533	0.01634 *
~ELS+VAS	204	-0.1911	1.685×10^6	0.006192	0.01867 *
~Age+VAS	205	0.1865	1.168×10^6	0.007404	0.02198 *
~PIL+MeDAS	210	0.1827	1.261×10^6	0.007948	0.02323 *
~Age+MNA	210	-0.1822	1.825×10^6	0.008141	0.02344 *
~GDS+MNA	213	-0.1747	1.892×10^6	0.01063	0.03013 *
~PSS+LSNS	213	-0.1734	1.89×10^6	0.01123	0.03139 *
~BMI+IPAQ	206	-0.1739	1.71×10^6	0.0124	0.03415 *
~JSS+STOPBANG	213	0.1671	1.341×10^6	0.01461	0.03965 *
~ERICE+VAS	204	0.1693	1.175×10^6	0.0155	0.04147 *
~PSS+ADL	213	0.1606	1.352×10^6	0.01897	0.04989 *
~ERICE+CRC	207	-0.1627	1.719×10^6	0.01917	0.04989 *
~ADL+IPAQ	213	-0.1571	1.864×10^6	0.02182	0.05603 *
~PIL+ADL	210	-0.1569	1.786×10^6	0.02296	0.05772 *
~UCLA+JSS	213	0.1556	1.36×10^6	0.02309	0.05772 *
~PHQ+MNA	213	-0.1539	1.858×10^6	0.02471	0.06095 *
~GDS+CRC	212	-0.1539	1.832×10^6	0.02502	0.06095 *
~ELS+ADL	209	-0.1528	1.754×10^6	0.02715	0.06531 *

Formula	n	Estimate	Statistic	p.Value	fdr
~OLQ+MeDAS	211	0.1447	1.339×10^6	0.03575	0.0849 *
~Age+CRC	209	-0.1448	1.742×10^6	0.03646	0.08551 *
~LSNS+STOPBANG	213	-0.143	1.841×10^6	0.03705	0.08584 *
~Age+PIL	207	-0.1413	1.687×10^6	0.04225	0.09568 *
~UCLA+MNA	213	-0.1393	1.835×10^6	0.0423	0.09568 *
~CRC+IPAQ	212	0.1382	1.369×10^6	0.04443	0.09932 *
~BRCS+CRC	212	0.129	1.383×10^6	0.06076	0.1342 *
~LSNS+IPAQ	213	0.1284	1.404×10^6	0.06143	0.1342 *
~ELS+JSS	209	-0.1274	1.715×10^6	0.06609	0.1427 *
~VAS+BMI	204	0.1219	1.242×10^6	0.08247	0.1761 *
~ELS+BMI	202	0.1217	1.207×10^6	0.08447	0.1783 *
~VAS+JSS	208	0.1184	1.322×10^6	0.08863	0.1851 *
~LSNS+MNA	213	0.1163	1.423×10^6	0.09055	0.187 *
~GDS+ADL	213	0.1149	1.426×10^6	0.09453	0.1931 *
~LSNS+JSS	213	-0.1134	1.793×10^6	0.0989	0.1999 *
~PSS+MeDAS	213	-0.1114	1.79×10^6	0.105	0.2079 *
~Age+PSS	210	0.1119	1.371×10^6	0.1058	0.2079 *
~OLQ+ERICE	205	0.1132	1.273×10^6	0.1061	0.2079 *
~VAS+IPAQ	208	-0.1101	1.665×10^6	0.1133	0.2196 *
~PIL+CRC	209	0.109	1.356×10^6	0.1162	0.2231 *
~ERICE+BMI	202	0.1093	1.224×10^6	0.1215	0.2288 *
~UCLA+IPAQ	213	-0.1061	1.781×10^6	0.1227	0.2288 *
~ELS+CRC	208	0.1073	1.339×10^6	0.1228	0.2288 *
~MeDAS+STOPBANG	213	-0.101	1.773×10^6	0.1419	0.2618 *
~Age+IPAQ	210	-0.101	1.699×10^6	0.1448	0.2634 *
~UCLA+MeDAS	213	-0.09986	1.771×10^6	0.1464	0.2634 *
~PIL+ERICE	204	-0.1019	1.559×10^6	0.147	0.2634 *

Formula	n	Estimate	Statistic	p.Value	fdr
~MeDAS+IPAQ	213	0.09773	1.453×10^6	0.1552	0.2753 *
~UCLA+ADL	213	0.09743	1.454×10^6	0.1565	0.2753 *
~LSNS+CRC	212	0.09669	1.434×10^6	0.1607	0.2801 *
~PHQ+LSNS	213	-0.0956	1.765×10^6	0.1644	0.284 *
~BRCS+ADL	213	-0.09384	1.762×10^6	0.1724	0.2951 *
~BRCS+JSS	213	-0.09241	1.759×10^6	0.179	0.3037 *
~Age+GDS	210	0.09069	1.403×10^6	0.1905	0.3203 *
~Age+UCLA	210	0.08803	1.408×10^6	0.2039	0.3399 *
~PIL+JSS	210	-0.08767	1.679×10^6	0.2058	0.34 *
~OLQ+ADL	211	-0.08568	1.7×10^6	0.2152	0.3514 *
~BRCS+VAS	208	-0.08607	1.629×10^6	0.2164	0.3514 *
~BRCS+MeDAS	213	0.08373	1.476×10^6	0.2236	0.3581 *
~ERICE+MNA	207	-0.08483	1.604×10^6	0.2243	0.3581 *
~ADL+CRC	212	-0.08216	1.718×10^6	0.2336	0.3699 *
~PSS+CRC	212	-0.08163	1.718×10^6	0.2366	0.3715 *
~BRCS+IPAQ	213	0.07994	1.482×10^6	0.2454	0.3813 *
~ADL+JSS	213	0.07969	1.482×10^6	0.2468	0.3813 *
~Age+STOPBANG	210	0.07786	1.423×10^6	0.2613	0.4004 *
~BRCS+ERICE	207	0.07656	1.365×10^6	0.2729	0.4148 *
~GDS+MeDAS	213	-0.07449	1.731×10^6	0.2792	0.4148 *
~BRCS+MNA	213	0.07448	1.491×10^6	0.2792	0.4148 *
~OLQ+IPAQ	211	0.0748	1.449×10^6	0.2794	0.4148 *
~VAS+MeDAS	208	0.07477	1.388×10^6	0.2831	0.4152 *
~GDS+IPAQ	213	-0.07373	1.729×10^6	0.2841	0.4152 *
~PSS+IPAQ	213	-0.07329	1.729×10^6	0.287	0.4162 *
~PHQ+CRC	212	-0.07175	1.702×10^6	0.2984	0.4295 *
~CRC+MNA	212	-0.07053	1.7×10^6	0.3067	0.4382 *

Formula	n	Estimate	Statistic	p.Value	fdr
~Age+OLQ	208	0.07076	1.394×10^6	0.3098	0.4393 *
~PSS+BMI	206	-0.06651	1.554×10^6	0.3422	0.4814 *
~Age+LSNS	210	-0.06554	1.645×10^6	0.3446	0.4814 *
~PSS+STOPBANG	213	0.06385	1.508×10^6	0.3538	0.4878 *
~MNA+IPAQ	213	0.06377	1.508×10^6	0.3543	0.4878 *
~ERICE+JSS	207	0.06436	1.383×10^6	0.3569	0.4878 *
~GDS+STOPBANG	213	0.06209	1.511×10^6	0.3672	0.4984 *
~PSS+JSS	213	0.05833	1.517×10^6	0.397	0.535
~LSNS+ERICE	207	-0.05564	1.561×10^6	0.4259	0.5698
~CRC+JSS	212	-0.0531	1.672×10^6	0.4418	0.587
~BMI+MeDAS	206	-0.0527	1.534×10^6	0.4519	0.5962
~ELS+ERICE	203	0.05087	1.323×10^6	0.4711	0.6173
~VAS+MNA	208	-0.04882	1.573×10^6	0.4838	0.6296
~LSNS+VAS	208	-0.04625	1.569×10^6	0.5071	0.653
~PHQ+ADL	213	0.04546	1.537×10^6	0.5093	0.653
~ADL+BMI	206	-0.04593	1.524×10^6	0.5121	0.653
~ELS+STOPBANG	209	0.04267	1.457×10^6	0.5396	0.6835
~UCLA+ERICE	207	0.04121	1.417×10^6	0.5555	0.699
~PHQ+ERICE	207	-0.03964	1.537×10^6	0.5707	0.7133
~LSNS+BMI	206	0.0363	1.404×10^6	0.6044	0.7482
~BRCS+STOPBANG	213	-0.03549	1.668×10^6	0.6065	0.7482
~ELS+IPAQ	209	0.0351	1.468×10^6	0.6139	0.7525
~Age+BRCS	210	-0.03345	1.595×10^6	0.6299	0.7671
~OLQ+BMI	204	0.03337	1.368×10^6	0.6356	0.7673
~GDS+ERICE	207	0.03259	1.43×10^6	0.641	0.7673
~Age+MeDAS	210	0.03226	1.494×10^6	0.6421	0.7673
~VAS+STOPBANG	208	0.03078	1.454×10^6	0.659	0.7817

Formula	n	Estimate	Statistic	p.Value	fdr
~ADL+MeDAS	213	-0.03008	1.659×10^6	0.6624	0.7817
~PIL+STOPBANG	210	-0.02971	1.589×10^6	0.6686	0.7841
~Age+JSS	210	0.02854	1.499×10^6	0.6809	0.7937
~PIL+BMI	203	0.02506	1.359×10^6	0.7227	0.8373
~GDS+BMI	206	0.02252	1.424×10^6	0.748	0.8579
~IPAQ+STOPBANG	213	-0.02127	1.645×10^6	0.7577	0.8579
~MNA+JSS	213	-0.02126	1.645×10^6	0.7577	0.8579
~UCLA+STOPBANG	213	-0.02118	1.645×10^6	0.7586	0.8579
~ADL+STOPBANG	213	0.01982	1.579×10^6	0.7737	0.8698
~Age+ELS	206	-0.01765	1.483×10^6	0.8012	0.8954
~BMI+JSS	206	0.01684	1.432×10^6	0.8101	0.8994
~PSS+ERICE	207	-0.01644	1.503×10^6	0.8141	0.8994
~ERICE+MeDAS	207	-0.01359	1.498×10^6	0.8459	0.9277
~Age+PHQ	210	-0.01317	1.564×10^6	0.8496	0.9277
~PHQ+MeDAS	213	-0.01223	1.63×10^6	0.8591	0.9328
~MNA+STOPBANG	213	0.01131	1.592×10^6	0.8697	0.9388
~OLQ+STOPBANG	211	-0.009972	1.581×10^6	0.8855	0.9462
~CRC+STOPBANG	212	-0.009524	1.603×10^6	0.8903	0.9462
~CRC+MeDAS	212	0.009433	1.573×10^6	0.8914	0.9462
~BRCS+BMI	206	-0.008853	1.47×10^6	0.8995	0.9495
~MeDAS+MNA	213	-0.008096	1.624×10^6	0.9065	0.9506
~UCLA+CRC	212	0.007756	1.576×10^6	0.9106	0.9506
~PHQ+STOPBANG	213	-0.007129	1.622×10^6	0.9176	0.9514
~ERICE+IPAQ	207	0.006556	1.469×10^6	0.9253	0.9514
~UCLA+BMI	206	0.00648	1.447×10^6	0.9263	0.9514
~Age+BMI	203	0.00513	1.387×10^6	0.9421	0.9565
~PHQ+BMI	206	0.00473	1.45×10^6	0.9462	0.9565

Formula	n	Estimate	Statistic	p.Value	fdr
~MeDAS+JSS	213	0.004632	1.603×10^6	0.9464	0.9565
~PHQ+IPAQ	213	-0.003882	1.617×10^6	0.9551	0.9601
~IPAQ+JSS	213	0.002273	1.607×10^6	0.9737	0.9737

n: sample size; fdr: false discovery rate. *: p-value < 0.05; **: p-value < 0.01; ***: p-value < 0.001.
